# The influence on perceptions of truthfulness of the emotional expressions shown when talking about failure

**DOI:** 10.5964/ejop.v11i1.877

**Published:** 2015-02-27

**Authors:** Shlomo David, Shlomo Hareli, Ursula Hess

**Affiliations:** a Graduate School of Management, University of Haifa, Haifa, Israel; b The Interdisciplinary Center for Research on Emotions, Haifa, Israel; c Humboldt-University, Berlin, Germany; University of South Wales, Newport, United Kingdom

**Keywords:** emotions, perceived truthfulness, organizational inquiry

## Abstract

The study aimed to assess whether showing emotion in an organizational inquiry into failure affects perceptions of truthfulness as a function of the match between the explanation of what caused the failure and the emotion expressed. Two web-based studies were conducted. Participants with work experience saw videos of an inquiry and rated the protagonist’s truthfulness. In both studies protagonists who expressed an emotion (anger or shame) were rated as less truthful than protagonists who expressed no emotion, regardless of what the failure was attributed to. In order to not confound effects of emotions with occupational stereotype effects only male protagonists were shown. Showing emotions when questioned is normal. Managers have to be aware of a tendency to count this against the employee. This is the only research focusing on the effects of showing emotions on perceptions of truthfulness in an organizational context.

In an organization things may go wrong and the reasons for this need to be ascertained, since the consequences of errors and failures can include wasted working hours, product disqualification, lowered customer satisfaction, damaged reputation of the firm, harmed relationships between the failing employee and managers and/or co-workers, injuries, and even the loss of lives (Kletz, 2001). Thus, in order to prevent repetition, organizations need to examine the reasons for the adverse events.

Yet, one condition for such an inquiry to be effective is that the employee(s) involved provide a full and accurate explanation of what happened. Under some circumstances, however, the employee may feel threatened by the inquiry, and as a result, withhold important information or provide a distorted explanation of what happened (Schlenker, Pontari, & Christopher, 2001; Schlenker & Weigold, 1992; Shaw, Wild, & Colquitt, 2003; Sigmon & Snyder, 1993; Snyder, Higgins, & Stucky, 1983). That is, the truthfulness of the employee(s) statements needs to be evaluated.

Research on truthfulness has generally focused on the verbal and nonverbal cues that distinguish between people who lie and those who tell the truth (see e.g., DePaulo et al., 2003; Masip, Sporer, Garrido, & Herrero, 2005; Sporer & Schwandt, 2006; Vrij, Granhag, & Porter, 2010) rather than on the cues that humans use to detect lies. Of the nonverbal cues that mark lies in the eye of the perceiver many are associated with emotional responding. Specifically, acting nervously is one of the cues that laypersons most systematically report as a cue to deception (Andrewartha, 2008; Taylor & Hick, 2007; Vrij & Semin, 1996) and nervous appearances (Kraut & Poe, 1980) as well as specific cues such as nervous gestures, changes in pitch and smiles are all used as indicators of deception (DePaulo, 1988; Riggio & Friedman, 1983; Zuckerman, DePaulo, & Rosenthal, 1981). Nervousness in this context is seen as a sign of guilt about lying or apprehension about being detected and punished (Davis, 1961).

By contrast, in the more specific context of victim testimony showing emotions has been found to increase believability for laypeople (Golding, Fryman, Marsil, & Yozwiak, 2003; Kaufmann, Drevland, Wessel, Overskeid, & Magnussen, 2003) and policemen (Bollingmo, Wessel, Eilertsen, & Magnussen, 2008) but not judges (Wessel, Drevland, Eilertsen, & Magnussen, 2006). Also the effect has been found to be stronger for individual judgments; when mock-juries discussed credibility, a neutral expression was favored as more truthful (Dahl et al., 2007). What is noteworthy in this context, is that when emotions enhance credibility at all, it is not the authenticity of the emotion – since a person who has experienced a trauma may well experience numbness and hence present flat affect – but the congruency with the normative expectations for the situation which seems to lead to perceptions of truthfulness. More generally, Vrij and Fischer (1997) propose that emotions increase credibility when they fit with the emotion rules for the situation such as showing despair when reporting on a traumatic event. In this same vein, Bond et al. (1992), note that when nonverbal behavior seems “off” or as they call it “fishy” it is perceived as a signal of deception.

However, it can be argued that anyone who wants to convince others of their position will be emotional to some degree (Fiske, 2004). In fact, one function of emotions in speech is to provide emphasis (Hamilton, Hunter, & Burgoon, 1990).

The present research aimed to assess whether presenting an argument designed to convince someone else is more believable when presented emotionally or in a neutral manner. In particular, we investigated whether statements made by an employee involved in a failure event regarding his role in the event, were believed more when presented neutrally or in an emotional manner.

Specifically, when observers interpret emotion expressions they tend to consider not only the physical changes in the face (such as the corners of the lips being turned up in a smile), but also the knowledge that they have about the person and the situation (Kirouac & Hess, 1999). Hence in a situation where the norm is for rational calm discussion, expressing emotions may elicit suspicion, because in the terms of Vrij and Fischer (1997) the emotion does not fit the emotion rule for the situation.

In sum, emotions that accompany a statement about an event possibly caused by the expresser can have two possible results. First, as was the case for victims (Bollingmo et al., 2008; Golding et al., 2003; Kaufmann et al., 2003) and complaints (Hareli et al., 2009), statements may be perceived as more truthful when accompanied by a congruent expression rather than an incongruent or neutral expression. In this context, shame would be congruent with admissions of responsibility for the event, whereas anger would be congruent with statements blaming an external source. Specifically, as suggested by appraisal theory (e.g., Frijda, 1986; Scherer, 1984), shame is elicited when the expresser perceives the undesirable outcome as caused by something internal to oneself. By contrast, anger is caused when attributing the outcome to someone else. People are aware of this connection between the emotions and their corresponding appraisals (Hareli & Hess, 2010) and may therefore interpret shame and anger as conveying admission of fault or as blaming others respectively. Thus, one expectation was that an interaction between type of statement and emotion will emerge such that when a verbal statement is in congruent with the “message” contained in the emotion shown by the employee, the employee will be believed more than when these messages are incongruent. However, admitting responsibility for the failure, in and of itself, may increase perceptions of truthfulness because people are motivated to protect themselves and when they act against this motivation they may be believed more (Jones, Davis, & Gergen, 1961; Kraut, 1978).

Second, if being emotional at all is perceived as an attempt to hide something or to manipulate the observer, then a calm, neutral presentation should be perceived as more truthful. Such a stance would also be more in line with organizational norms and hence may have specific effects on organizational consequences. In other words, employees may attempt to regulate any emotions – even those that are congruent with their verbal message, because they are in conflict with organizational display rules that demand neutrality. That is, they would engage in emotional labor to conform to organizational rules (Hochschild, 1983).

Finally, one may expect that when workers refuse to take responsibility for the failure, they will be believed less. This is in line with findings on self-serving explanations, which suggest that deflecting guilt by blaming others reduces believability (Jones et al., 1961; Kraut, 1978).

## The Present Research

Two studies were conducted. In Study 1 an actor played the role of an employee who was involved in a situation where a failure occurred which may have been due to his action, in Study 2, two actors were employed. In both studies, the actor(s) presented their statements either neutrally or while showing anger or shame. The employee’s statement was either ambiguous, a rejection of responsibility or an admission of responsibility.

The mere fact that an organizational inquiry had been conducted may be seen by participants as an indication that there is external evidence for the employee’s guilt. Specifically, they may, as is the case in criminal proceedings (cf. Babcock, 2005), assume that “where there is smoke there is fire” and that an innocent person would be diverted before it came to an inquiry. Hence in Study 1, participants received a preliminary report that commented on the fact that the employee was known to be reliable; in Study 2, participants were provided either with a preliminary report that specifically corroborated the employee’s claims or with unrelated information about the organization.

## Study 1

### Method

#### Participants

A total of 416 (254 men) individuals with a mean age of 47 years (*SD* = 10.94), participated in a web based study. Fifty-five percent of the participants reported direct personal experience with organizational inquiries. Participants were recruited via email from a list of over 1000 people including the pool of alumni of the graduate school of management of the University of Haifa and friends of the authors with work experience. Response rate was around 10% with no difference in response rate across experimental conditions.

#### Stimulus material

A video showed part of a staged organizational inquiry that was based on an actual failure event. Specifically, a software technician in a company that develops software for electronic commerce is responsible for an upgrade of a customer's system. Following that upgrade, the system crashed and was inoperative for a few hours, which caused considerable damage to the customer. The goal of the inquiry was to understand what caused the failure. Participants were told that the short video they would be watching was an excerpt in which the employee answers the question "what in your opinion caused the problem?". Prior to seeing the video, participants received information from a “preliminary report” that commented on the fact that the employee was known to be reliable.

The employee responded by (a) a statement taking responsibility where the employee states that he assumed this to be just a routine installation and hence decided to skip the required diagnostics prior to installation or (b) a statement blaming someone else where he states that this was routine installation and his supervisor told him to skip the required diagnostics, at the time he did not question this, but now he thinks that this was the cause of the problem c) gives an ambiguous answer, stating that he really does not know what happened, he had followed the relevant instructions and done what he always did in similar situations but this time the system crashed. In each case, he further speculated that the fault was maybe a bug in the customer's system.

A professional actor played the employee and answered the question in either a neutral manner or expressing anger or shame. This resulted in a 3 (type of answer: blames other, takes responsibility, ambiguous answer) x 3 (employee’s emotion: anger, shame, neutrality) design.

#### Procedure

List members received one of three different web-links that included the stimulus material and questionnaire for the experiment. This material contained information describing the organization and that the employee in question was generally known to be reliable.

Upon entering the web-site, participants first read the context information and then saw the video. Following the video, they answered a series of questions. All ratings were made on 7-point scales ranging from 0 = "not at all" to 6 = "to a large extent."

#### Dependent Variables

After watching the video, participants answered a series of questions. These also included questions regarding their understanding of the interview and their perception of the employee as well as about their emotional reactions to the event. These data will not be discussed in the framework of this report.

*Truthfulness*. Participants were asked whether they believed the employee.

*Consequences*. Participants were asked whether they would recommend firing the individual as well as whether they would consider promoting the individual in the future.

*Manipulation check*. Following these questions participants were asked a couple of questions about their perception of the employee, which included a manipulation check. For this they rated the emotion shown by the employee on the scales: relaxed, anger, shame and guilt. All scales ranged from 0 – “not at all” to 6 – “to a large extent”.

### Results

#### Manipulation check

A series of 3 (type of answer) x 3 (emotion expression) analyses of variance were conducted on the emotion ratings. Significant main effects emerged for all four emotion scales, Anger: *F*(2,260) = 147.99, *p* < .0001, η_p_^2^ = .53, Shame: *F*(2,260) = 62.47, *p* < .0001, η_p_^2^ = .33, Guilt: *F*(2,260) = 19.03, *p* < .0001, η_p_^2^ = .13, Relaxed: *F*(2,259) = 80.93, η_p_^2^ = .39, *p* < .0001, (see Table 1 for means and standard deviations).

**Table 1:** Manipulation Check

Perceived employee emotion	Conditions					
	Anger		Shame		Neutral	
	Mean	*SD*	Mean	*SD*	Mean	*SD*
Study 1						
Angry	5.00^a^	1.29	2.00^b^	1.32	1.98^b^	1.48
Ashamed	1.91^a^	1.68	4.34^b^	1.44	2.36^a^	1.53
Guilty	2.52^a^	1.91	3.89^b^	1.52	2.68^a^	1.71
Relaxed	1.24^a^	1.24	2.92^b^	1.42	3.70^c^	1.35
Study 2						
Angry	4.90^a^	1.17	1.69^b^	1.21	1.84^b^	1.36
Ashamed	2.47^a^	1.76	4.70^b^	1.09	2.24^a^	1.62
Relaxed	1.10^a^	1.21	2.64^b^	1.36	3.70^c^	1.22

*Note.* Subscripts based on Fisher LSD tests at *p <*.05. Numbers with different subscripts differ at *p < .*05.Higher numbers represent higher ratings.

Overall the manipulation check confirmed that the actor was perceived as intended. Specifically, in the anger condition the actor was perceived as most angry, in the shame condition the actor was perceived as most ashamed and guilty and in the neutral condition the actor was perceived as low in anger, shame and guilt as well as most relaxed.

For guilt and relaxed significant main effects of type of answer emerged, guilt: *F*(2,260) = 17.27, *p* < .001, η_p_^2^ = .12, relaxed: *F*(2,259) = 5.36, *p* = .005, η_p_^2^ = .04, such that when accepting responsibility for the failure the employee was perceived as more guilty (*M* = 3.84, *SD* = 1.52) and relaxed (*M* = 2.99, *SD* = 1.69) than when blaming someone else (guilt: *M* = 2.57, *SD* = 1.89, relaxed: *M* = 2.49, *SD* = 1.65) or giving an ambiguous answer (guilt: *M* = 2.66, *SD* = 1.76, relaxed: *M* = 2.62, *SD* = 1.68) for which perceptions did not differ. The fact that when assuming responsibility for the event the employee was perceived as experiencing more guilt is congruent with the notion that this verbal statement is equivalent to an admission of guilt. It is possible that participants considered that an admission of guilt should have a positive, unburdening, effect and hence assumed him to be also more relaxed than when he seemed to be fending off an accusation.

#### Truthfulness

A 3 (answer type) x 3 (emotion expression) analysis of variance was conducted (see Table 2 for means and standard deviations). Main effects of answer type, *F*(2,258) = 9.08, *p* < .0001, η_p_^2^ = .07, and emotion expression, *F*(2,258) = 8.53, *p* < .0001, η_p_^2^ = .06, emerged. The answer type by emotion interaction was not significant, *F*(2,258) = 0.58, *p* = .676, η_p_^2^ = .009. Overall, participants believed the employee more when he did not show any emotion than when he showed either anger or shame for which there was no difference. They also believed the employee more when he took responsibility than when he blamed someone else or gave an ambiguous answer for which there was no difference.

#### Consequences

Three (type of answer) x 3 (emotion expression) analyses of variance were conducted on recommendations to fire the employee now and the possibility to promote the employee in the future. Neither factor had an impact on the immediate consequences for the employee. By contrast, both a main effect of emotion, *F*(2,256) = 15.14, *p* < .0001, η_p_^2^ = .11, and of type of answer emerged, *F*(2,256) = 3.75, *p* = .025, η_p_^2^ = .03, for intentions to promote the employee in the future. The pattern of results was consistent with the perceptions of the employee’s truthfulness. That is, participants were more likely to consider promoting the employee when he showed a neutral expression rather than shame or anger as well as when he admitted guilt rather than blamed someone else or gave an ambiguous answer.

### Discussion

Participants believed the employee most when he took verbally responsibility for the failure or when he showed a neutral expression. The same pattern was obtained for intentions to promote the employee in the future. The effects for answer type are congruent with the notion that admitting responsibility – which in this case is a direct admission of guilt with regard to the failure – increases perceptions of truthfulness congruent with the “ulterior motive rule” that self-serving explanations – in this case those that deflect guilt by blaming someone else or giving an ambiguous answer – are believed less (Jones et al., 1961; Kraut, 1978). Consistent with Kraut (1978) there was also a (non-significant) trend for ambiguous answers to be believed less.

Further, individuals who admit guilt should engender more trust with regard to their future behavior. Specifically, Frank (1988) maintains that guilt is a moral emotion that signals not only understanding of a misdeed but also intentions to do better in the future. Hence such a person should be more likely to be promoted in the future.

Neutral expressions were perceived as most indicative of truth. This may suggest that participants perceived the emotion expression as a sign of nervousness indicative of lying (Andrewartha, 2008; Kraut & Poe, 1980; Taylor & Hick, 2007) rather than as congruent with the message. Also, in this study we explicitly mentioned that the employee was known to be reliable. That is, there was good reason for the participants to believe him. Hence, it is possible that in an organizational context where the norm is for emotional neutrality (Ashforth & Humphrey, 1995), unwarranted emotion when the truth has largely been established, is a norm violation which elicits suspicion. We therefore conducted a second study, in which we varied whether information about the credibility of the employee was provided. We also used two different actors to assure that effects of credibility are not idiosyncratic to the actor.

## Study 2

### Method

#### Participants

A total of 422 (159 men) individuals with a mean age of 32.1 (*SD* = 10.96) years participated in a web based study. A snowball system was employed where employees of various companies were contacted by email and asked to send the email on to other employees they know.

The same overall method and procedure was employed. However, for this study two actors performed according to the same script. In the manipulation check guilt was not included. We also added the question whether the employee seemed credible in general to assess possible effects of personality attributions. As this question correlated r = .78 with the believability question, we combined the two.

Two hundred-seventeen participants were provided with information about a preliminary inquest in which the same information as provided by the employee in the video was given by another employee. The remaining participants received general information about the company. To assure that the information would be read, questions about its content were asked. All participants gave correct answers.

### Results

#### Manipulation check

A series of 3 (type of answer) x 3 (emotion expression) 2 (credibility information) analyses of variance were conducted on the emotion ratings. Significant main effects emerged for all three emotion scales, Anger: *F*(2,404) = 289.86, *p* < .0001, η_p_^2^ = .59, Shame: *F*(2,404) = 122.01, *p* < .0001, η_p_^2^ = .38, Relaxed: *F*(2,404) = 145.71, *p* < .0001, η_p_^2^ = .42, (see Table 1 for means and standard deviations). Overall the manipulation check confirmed that the actor was perceived as intended. Specifically, in the anger condition the actor was perceived as most angry, in the shame condition the actor was perceived as most ashamed and in the neutral condition the actor was perceived as low in anger, and shame as well as most relaxed.

For anger and shame a significant main effect of type of answer emerged, *F*(2,404) = 4.68, *p* = .01, η_p_^2^ = .02, and, *F*(2,404) = 12.05, *p* < .0001, η_p_^2^ = .06, such that when blaming someone else the employee seemed more angry (*M* = 3.06, *SD* = 1.93) and less ashamed (*M* = 2.68, *SD* = 2.02) than when accepting responsibility (anger: *M* = 2.58, *SD* = 1.90, shame: *M* = 3.53, *SD* 1.76) or giving an ambiguous answer (anger: *M* = 2.70, *SD* = 1.95, Shame: *M* = 3.14, *SD* = 1.76) for which perceptions did not differ.

For shame an emotion x type of answer interaction emerged, *F*(4,404) = 3.21, *p* = .013, η_p_^2^ = .03, such that when the actor showed shame, there was no significant difference in the shame ratings as a function of type of answer (*M* = 4.70, *SD* = 1.09). However, when the actor showed anger or neutrality he was perceived as experiencing less shame when attributing the blame to someone else (anger: *M* = 1.91, *SD* = 1.66, neutral: *M* = 1.37, *SD* = 1.42) then when taking responsibility (anger: *M* = 2.87, *SD* = 1.77, neutral: *M* = 2.83, *SD* = 1.62) or giving an ambiguous answer (anger: *M* = 2.64, *SD* = 1.74, neutral: *M* = 2.44, *SD* = 1.49). This interaction seems to reflect a context effect, where knowledge about the answer impacts on perception of shame, but only when shame is not actually shown. In this case, participants seemed to attribute lower levels of shame, when they thought the actor has no reason to feel shame.

Overall, the manipulation check confirmed, that the actors seemed to express the most anger in the anger condition, the most shame in the shame condition and neutrality in the neutral condition.

#### Truthfulness

A 3 (answer type) x 3 (emotion expression) x 2 (credibility information) analysis of variance was conducted (see Table 2 for means and standard deviations). Main effects of answer type, *F*(2,404) = 17.15, *p* < .0001, η_p_^2^ = .08, emotion expression, *F*(2,404) = 30.20, *p* < .0001, η_p_^2^ = .13, and credibility information, η_p_^2^ = .13, *F*(1,404) = 5.03, *p* = .025, η_p_^2^ = .01, emerged. The interaction between emotion and credibility information was significant, *F*(2,404) = 3.55, *p* = .03, η_p_^2^ = .02, but no further significant interaction effects emerged.

Overall, as in Study 1, participants believed the employee more when he took responsibility than when he blamed someone else or gave an ambiguous answer, for which there was no difference. Also as in Study 1, the employee was believed more when he showed neutrality then when he showed either emotion, for which there was no difference. Credibility information had an effect such that when participants were told that the employee gave the same answer as another employee they believed the employee more (*M* = 3.69, *SD* = 1.38) than when they received information about the organization (*M* = 3.44, *SD* = 1.32).

As expected, an interaction between emotion and credibility information emerged. Credibility information only had a modulating effect for anger, such that participants believed the employee even less when he showed anger and no credibility information was given (*M* = 2.74, *SD* = 1.15). When credibility information was given, participants believed the employee somewhat more (*M* = 3.46, *SD* = 1.49), albeit less than when he showed a neutral expression. For the neutral and the shame expression credibility made no difference. Thus, credibility information had only a limited moderating effect. In fact, it seems that if the employee shows any emotion, as in Study 1, this is interpreted as a suspicious behavior, but this is aggravated when the employee shows anger without external evidence that he says the truth. In this case it seems that the anger expression is seen as a defensive effort to distract from guilt.

#### Consequences

Three (type of answer) x 3 (emotion expression) x 2 (credibility information) analyses of variance were conducted on recommendations to fire the employee now and the possibility to promote the employee in the future. In contrast to Study 1, main effects of emotion, *F*(2,404) = 17.15, *p* < .0001, η_p_^2^ = .08, type of answer, *F*(2,404) = 4.73, *p* = .009, η_p_^2^ = .02 and credibility information, *F*(1,404) = 30.80, *p* < .0001, η_p_^2^ = .07, emerged for intentions to fire (see Table 2).

**Table 2:** Means and Standard Deviations as a Function of Emotional Expression and Answer Type for Studies 1 and 2

	Believe answer		Fire		Promote	
	Mean	*SD*	Mean	*SD*	Mean	*SD*
Study 1						
*Answer type*						
Blames other	3.50_a_	1.47	3.28_a_	1.52	2.21_a_	1.49
Takes responsibility	4.09_b_	1.42	3.23_a_	1.46	2.66_b_	1.48
Ambiguous answer	3.19_a_	1.51	3.46_a_	1.49	2.17_a_	1.33
*Emotion expression*						
Anger	3.31_a_	1.61	3.53_a_	1.42	2.21_a_	1.49
Shame	3.36_a_	1.57	3.36_a_	1.47	1.86_a_	1.34
Neutral	4.10_b_	1.21	3.08_a_	1.48	2.97_b_	1.28
Study 2						
*Answer type*						
Blames other	3.41_a_	1.43	2.22_a_	1.52	1.53_a_	1.33
Takes responsibility	4.15_b_	1.53	2.70_b_	1.67	1.37_a_	1.58
Ambiguous answer	3.21_a_	1.43	2.26_a_	1.61	1.45_a_	1.26
*Emotion expression*						
Anger	3.08_a_	1.62	2.72_a_	1.63	1.30_a_	1.40
Shame	3.41_a_	1.48	2.67_a_	1.53	1.20_a_	1.14
Neutral	4.24_b_	1.19	1.82_b_	1.42	2.11_b_	1.23
*Credibility information*						
Information	3.71_a_	1.58	2.00_a_	1.50	1.59_a_	1.32
No Information	3.48_a_	1.45	2.76_b_	1.57	1.52_a_	1.32

*Note.* Subscripts based on Fisher LSD tests at *p <*.05. Numbers with different subscripts differ at *p < .*05.Higher numbers represent higher ratings.

Specifically, when the employee remained neutral, participants were less likely to recommend firing him, than when he showed an emotion. When the employee accepted responsibility participants were more likely to recommend that he should be fired than when he blamed someone else or gave an ambiguous answer. When participants received information about the organization (*M* = 2.76, *SD* = 1.57, they were more likely to recommend firing then when they received information about the credibility of the employee’s claim (*M* = 2.00, *SD* = 1.50).

For the intent to promote the employee, only a main effect of emotion, *F*(2,404) = 22.81, *p* < .0001, η_p_^2^ = .10, emerged. The pattern of results was consistent with the perceptions of the employee’s truthfulness. That is, participants were more likely to consider promoting the employee when he showed a neutral expression rather than shame or anger.

### Discussion

Overall, Study 2 replicated the finding from Study 1 that showing a neutral expression was perceived as more indicative of truthfulness than showing an emotion, regardless of type of emotion. Further, taking responsibility is perceived as more likely to be truthful than blaming someone else or being ambiguous about the cause. Specific information on the credibility of the employee interacted only weakly with emotional expression, such that anger was seen as more indicative of truthfulness when accompanied by information that supported the employee’s statement.

## General Discussion

The present research had the goal to assess the impact of showing an emotion while being asked about a failure event for the perceived truthfulness of the report. For this we manipulated the emotion shown (anger, shame, and no emotion), the type of response given (taking responsibility, blaming someone else, and staying vague about the cause) as well as in Study 2 additional information about the truthfulness of the statement.

As one may expect, the type of answer had an effect on perceived truthfulness. Specifically, participants believed the employee more when he took responsibility. This is in line with general observations from attribution theory that self-serving explanations – in this case those that deflect guilt by blaming someone else or giving an ambiguous answer – are believed less (Jones et al., 1961; Kraut, 1978). However, there was no interaction between answer type and emotion shown. That is, independent of whether the employee gave a more or less believable verbal statement, the emotion shown had the same effect.

Specifically, the present research showed in two studies that an employee who did not show emotions was perceived as more truthful than one who did. Interestingly, knowledge about the likely credibility of employee (Study 2) had only an effect when the employee showed anger, such that the negative effect of emotion expression on credibility was aggravated when there was no additional evidence that he says the truth. In this case it seems that the anger expression is seen as a defensive effort to distract from guilt. These effects also generalized to decisions to promote the employee in the future. Recommendations to fire the employee were only affected by the manipulations in Study 2, but here also the overall recommendation was in line with the notion that the employee who showed no emotion was less likely to be fired.

Overall, these findings converge to support the notion that in a business context, showing an emotion when interrogated about a failure event is perceived as indicative of lack of truthfulness. The most likely interpretation is that being emotional is seen as a sign of nervousness in the case of shame and maybe a sign of defensiveness in the case of anger. The latter is suggested by the effect of external credibility information when anger is shown. When there is external credibility information, participants rated the employee as somewhat more truthful, which suggests that in this case the anger was interpreted as a righteous outrage at being accused. In the absence of such specific information (Study 1) anger had the same effect as shame.

The lack of differential attributions based on the type of emotion is in and of itself interesting. One could argue that anger – which is related to the core relational theme of being insulted (Lazarus, 1991) should be interpreted as a sign of righteous outrage, whereas shame, which is typically shown by someone guilty of some transgression (Lazarus, 1991), should signal guilt. Hence, the employee who shows anger should be given more credibility when he declares himself to be innocent – especially when other external corroborating information is not provided, as was shown by Hareli et al. (2009) for customers who complain, whereas the person who shows shame should be considered more truthful when taking responsibility and less truthful when blaming someone else. However, not only is the emotion by answer type interaction not significant, but the pattern of means is not congruent with such an interpretation.

Thus, in the present research the type of emotion shown had basically no influence on the perception of truthfulness. This finding is in line with the notion that emotional expressions -- and the lack of expressions – have to be congruent with prevailing norms to support perceptions of honesty (Vrij & Fischer, 1997). As mentioned above, prevailing workplace norms tend to emphasize emotional neutrality, objectivity and professionalism (cf. Ashforth & Humphrey, 1995; Weber, 1968), hence a calm, neutral stance is perceived as most truthful.

One important limitation of the study was that we used only male actors. This was done for several reasons. First of all, many workplaces are still gendered in that one sex is considered generally more apt in that domain than the other. Second, and more importantly, there is evidence that emotion expressions shown by men and women are not interpreted in quite the same way. Thus, Hess, Adams, and Kleck (2004, 2005, 2009) showed that anger and happiness are perceived differently when shown by men and women and that this interacts with perceived dominance and affiliation. Brescoll and Uhlmann (2008) showed that when women in a business context show anger, this is attributed to their personality and not as a reaction to the situation. Given these considerations, gender and emotion manipulation would likely have given confounded effects. Future research should be conducted to assess the role of emotion expression for perceived truthfulness for female employees. Another limitation of the present research was that we limited the organizational failure to one context, which may have had a unique impact on our results due to stereotypes that people have about this type of occupation. Likewise, we had only one level of damage caused by the failure. Future research should assess the same research question in other occupational domains and also manipulate the severity of the failure.

In sum, the present research shows that expressing emotions when making important statements about failure events can lead to impressions of lack of truthfulness. This is an important finding as people often express emotions when wanting to convince others (Fiske, 2004) or to provide emphasis in speech (Hamilton et al., 1990). For the manager this means that emotional utterances should not be readily discarded but the whole situation – and the degree that it engenders emotionality in the employee – needs to be taken into account. Using participants with work experience is of particular importance in this context. On a more general level, using video stimuli to test the social perception of emotions proves as a promising strategy.
